# Effect of Dexmedetomidine on Heart Rate-Corrected QT and T_peak_–T_end_ Intervals During Robot-Assisted Laparoscopic Prostatectomy With Steep Trendelenburg Position: A Prospective, Randomized, Double-Blinded, Controlled Study

**DOI:** 10.1097/MD.0000000000003645

**Published:** 2016-05-13

**Authors:** Na Young Kim, Dong Woo Han, Jae Chul Koh, Koon Ho Rha, Jung Hwa Hong, Jong Min Park, So Yeon Kim

**Affiliations:** From the Department of Anesthesiology and Pain Medicine (NYK, DWH, JCK, JMP, SYK); Anesthesia and Pain Research Institute (NYK, DWH, JCK, SYK); Department of Urology, Urological Science Institute (KHR); Department of Research Affairs, Biostatistics Collaboration Units (JHH), Yonsei University College of Medicine, Seoul, Republic of Korea.

## Abstract

Intraperitoneal insufflation of carbon dioxide may affect the sympathetic activity that leads to changes in ventricular repolarization. This in turn can result in changes of heart rate-corrected QT (QTc) interval and Tpeak–Tend (Tp-e) interval. Dexmedetomidine is a highly selective α2-receptor agonist and has potential antiarrhythmic properties. This prospective, randomized, double-blinded, controlled study evaluated the effects of dexmedetomidine administration on QTc and Tp-e intervals during robot-assisted laparoscopic prostatectomy with steep Trendelenburg position.

Fifty patients scheduled for robot-assisted laparoscopic prostatectomy randomly received either a continuous infusion of dexmedetomidine at a rate of 0.3 μg/kg/hour, from anesthetic induction until the end of the Trendelenburg position (dexmedetomidine group; n = 25), or the same volume of normal saline (control group; n = 25). Anesthesia was maintained with sevoflurane and remifentanil. The primary and secondary goals were to evaluate the effect of dexmedetomidine on the QTc and Tp-e interval changes. Mean arterial pressure, heart rate, end-tidal CO_2_, and end-tidal sevoflurane concentrations were assessed as well.

Forty-seven patients (94%) completed the study. Dexmedetomidine significantly attenuated QTc interval prolongation and reduced the Tp-e interval, even though the baseline values of the QTc and Tp-e intervals were similar between the 2 groups (*P*_Group × Time_ = 0.001 and 0.014, respectively). Twenty-two patients (96%) in the control group and 13 (54%) in the dexmedetomidine group had QTc interval prolongation of >20 ms from the baseline value during surgery (*P* = 0.001). The maximum QTc interval prolongation from the baseline value during surgery was 46 ± 21 ms in the control group and 24 ± 21 ms in the dexmedetomidine group (mean ± SD, *P* = 0.001). Mean arterial pressure and heart rate were comparable between the groups.

Continuous infusion of dexmedetomidine at a rate of 0.3 μg/kg/hour significantly attenuated the QTc interval prolongation induced by CO_2_ pneumoperitoneum with steep Trendelenburg position. Furthermore, dexmedetomidine reduced the Tp-e interval. Thus, dexmedetomidine administration may be effective for patients who are susceptible to the development of ventricular arrhythmia during robot-assisted laparoscopic prostatectomy.

## INTRODUCTION

Intraperitoneal insufflation of carbon dioxide (CO_2_) is the most commonly used method for adequate surgical access during laparoscopic surgery. However, CO_2_ pneumoperitoneum may influence autonomic nervous activity, resulting in sympathetic hyperactivity and alterations in circulatory hemodynamics due to increased intraabdominal pressure.^1–5^ In fact, cardiac arrhythmias such as sinus tachycardia, ventricular tachycardia, and asystole have been reported during CO_2_ pneumoperitoneum.^6^ Robot-assisted laparoscopic prostatectomy requires a steep Trendelenburg position in addition to CO_2_ pneumoperitoneum. The combination of the 2 may induce significant cardiovascular changes, and an increase in head-down angle may further augment these changes.^7,8^

The stimulation of sympathetic activity provokes changes in cardiac repolarization,^9,10^ which can result in changes in the values of the indexes of ventricular repolarization, such as heart rate (HR)-corrected QT (QTc) interval and Tpeak–Tend (Tp-e) interval.^11–14^ Prolongation of the QTc and Tp-e intervals can lead to malignant ventricular arrhythmias such as torsades de pointes and even sudden cardiac death.^14–17^ QTc interval prolongation was reported in laparoscopic cholecystectomy, and the prolongation was significantly greater in the elderly than in the younger patients.^18^ Most patients who undergo prostatectomy are elderly with various comorbidities. However, no study has evaluated the changes in QTc and Tp-e intervals during robot-assisted laparoscopic prostatectomy.

Dexmedetomidine is a highly selective α2-receptor agonist that has sympatholytic, analgesic, and sedative properties.^19^ Apart from these properties, dexmedetomidine has potential antiarrhythmic properties and was reported to be effective in the prevention and treatment of perioperative tachyarrhythmia in congenital cardiac surgery.^20,21^ Furthermore, dexmedetomidine attenuated the QTc interval prolongation during tracheal intubation and spinal anesthesia.^22,23^

Thus, in this randomized, double-blind, placebo-controlled study, we evaluated the effect of dexmedetomidine on QTc and Tp-e intervals during robot-assisted laparoscopic prostatectomy with steep Trendelenburg position.

## METHODS

After acquiring approval from the Institutional Review Board and Hospital Research Ethics Committee of Severance Hospital, Yonsei University Health System, Seoul, Korea (IRB protocol No. 4-2015-0337), clinical trial registration was obtained at http://clinicaltrials.gov (registration No. NCT02536014). Between August, 2015 and January, 2016, 50 male patients with an American Society of Anesthesiologists physical status of I/II who were scheduled to undergo robot-assisted laparoscopic prostatectomy were enrolled, after written informed consents were obtained from all the patients. Patients were excluded if any of the following criteria were present: emergency surgery; preoperative electrocardiography (ECG) abnormalities, including a QTc interval of >440 ms, ventricular conduction abnormalities, or arrhythmias; history of cardiac disease such as pacemaker insertion, unstable angina, congestive heart failure, or valvular heart disease; use of antiarrhythmic agents or medications other than antihypertensive agents that are known to prolong the QTc interval; abnormal levels of preoperative serum electrolytes; concomitant hepatic or renal failure; any allergy or hypersensitivity to α2-adrenergic agonists; and neurological or psychiatric impairment.

The patients were randomly allocated to either the dexmedetomidine group (n = 25) or the control group (n = 25) according to the computer-generated random numbers (http://www.random.org). In the dexmedetomidine group, dexmedetomidine (100 μg/mL in a 2 mL-vial; Hospira Worldwide, Seoul, Korea) was administered at a rate of 0.3 μg/kg/hour, from anesthetic induction until the end of the Trendelenburg position. Dexmedetomidine 200 μg was diluted with normal saline to a total volume of 50 mL in order to make a concentration of 4 μg/mL. The control group received volume-matched normal saline infusion as placebo. Dexmedetomidine or normal saline was prepared by an anesthesiologist who did not participate in the data collection. The investigator, surgeons, attending anesthesiologist, recovery nurses, and patients were blinded to the group allocation.

Upon arrival to the operating room, 0.1 mg of glycopyrrolate was given intravenously as a premedication. In all the patients, ECG, oxygen saturation analysis, noninvasive arterial blood pressure monitoring, and bispectral index monitoring (Aspect A-2000; Aspect Medical System Inc., Newton, MA) were performed. Anesthesia was induced with propofol 1.5 to 2 mg/kg, remifentanil 0.5 μg/kg, and rocuronium 1.2 mg/kg. Mechanical ventilation was maintained at a tidal volume of 8 to 10 mL/kg and a positive end-expiratory pressure of 5 cm H_2_O. The respiratory rate was adjusted to maintain the end-tidal CO_2_ (EtCO_2_) at 35 to 45 mm Hg in 50% O_2_/air. After induction, a radial artery catheter was placed and arterial pressure was continuously monitored. Anesthesia was maintained with sevoflurane (0.7–1.2 age-adjusted minimal alveolar concentration) and remifentanil (0.02–0.1 μg/kg/min) by targeting bispectral index scores between 40 and 60.

The QT and Tp-e intervals from continuous ECG monitoring in lead V5 were collected by using the LabChart software (Pro Version 7: AD Instruments, CO., Sydney, Australia) and a data acquisition system (PowerLab; AD Instruments). The QT (onset of the QRS complex to the end of the T wave) and Tp-e intervals (from the peak to the end of the T wave) were measured at the following 9 separate time points automatically via lead V5: before induction of anesthesia in the supine position (Baseline); 10 minutes after tracheal intubation (Intu-10 min); immediately after steep Trendelenburg position with CO_2_ pneumoperitoneum (T-on); 30; 60; and 90 minutes after steep Trendelenburg position with CO_2_ pneumoperitoneum (T-30 min, T-60 min, and T-90 min); immediately after a supine position with CO_2_ desufflation (T-off); at the end of surgery (Surgery end); and 60 minutes after a supine position with CO_2_ desufflation in the postanesthetic care unit (T-off 60 min). If the automatic measurement did not correctly detect the earliest onset of the QRS complex or the end of the T wave, the QT and Tp-e intervals were measured manually by using the cursor from the earliest onset of the QRS complex or the highest amplitude of the T wave to the latest end of the T wave, where its terminal limb joined the baseline.^23^ The recorded measurements of QT and Tp-e intervals were averaged from 4 successive beats. The QT interval was corrected for HR by using the Bazett formula,

 
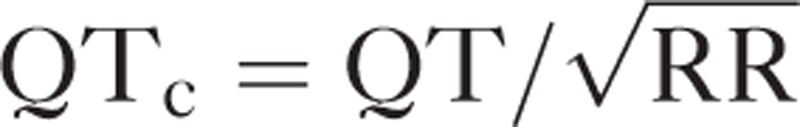


All the QT and Tp-e intervals were measured and analyzed by one of the authors, who was blinded to the group allocation.

Hemodynamic parameters including mean arterial pressure (MAP) and HR were collected at the same 9 separate time points as those for the QT and Tp-e intervals. EtCO_2_ and end-tidal sevoflurane concentrations were recorded at 7 separate time points from 10 minutes after tracheal intubation until the end of surgery. Hypotension (MAP <60 mm Hg) and bradycardia (HR <40 beats/min) were treated with intravenous ephedrine at 4-mg increments and atropine 0.5 mg, respectively. The CO_2_ insufflator was used to create a 20 mm Hg intraabdominal pressure in the supine position. Then, intraabdominal pressure was reduced to 15 mm Hg and maintained during the 29° Trendelenburg position.

### Statistical Analysis

The primary outcome of this study was the evaluation of the effect of dexmedetomidine on the QTc interval changes during robot-assisted laparoscopic prostatectomy with steep Trendelenburg position. In a preliminary study with 10 patients, the mean ± standard deviation (SD) of the QTc intervals at 60 minutes after steep Trendelenburg position with CO_2_ pneumoperitoneum was 389.8 ± 24.9 ms. Based on a previous study, a 25-ms reduction in the QTc interval could be considered to be clinically relevant.^22^ With a significant level of 5% and a statistical power of 90%, 22 subjects were required in each group. Considering a possible 10% dropout rate, 25 patients were enrolled in each group.

All the values were expressed as mean ± SD, median (range), or number of patients (proportion). Parametric data were analyzed by using the independent *t* test, and nonparametric data were analyzed by using the Mann–Whitney *U* test. Categorical variables were evaluated by using the χ^2^ test or Fisher exact test when appropriate. Repeatedly measured variables such as QTc interval, Tp-e interval, MAP, HR, EtCO_2_, and end-tidal sevoflurane concentration were analyzed by using a linear mixed model with group, time, and group-by-time as fixed effects. When the interaction of group, time, and group-by-time showed statistical significance, a post hoc analysis was performed with Bonferroni correction to adjust for multiple comparisons. A *P* value of <0.05 was regarded as statistically significant. Statistical analyses were performed by using the SAS version 9.2 software (SAS Inc., Cary, NC) and IBM SPSS Statistics 20 (SPSS Inc., Chicago, IL).

## RESULTS

**FIGURE 1:**
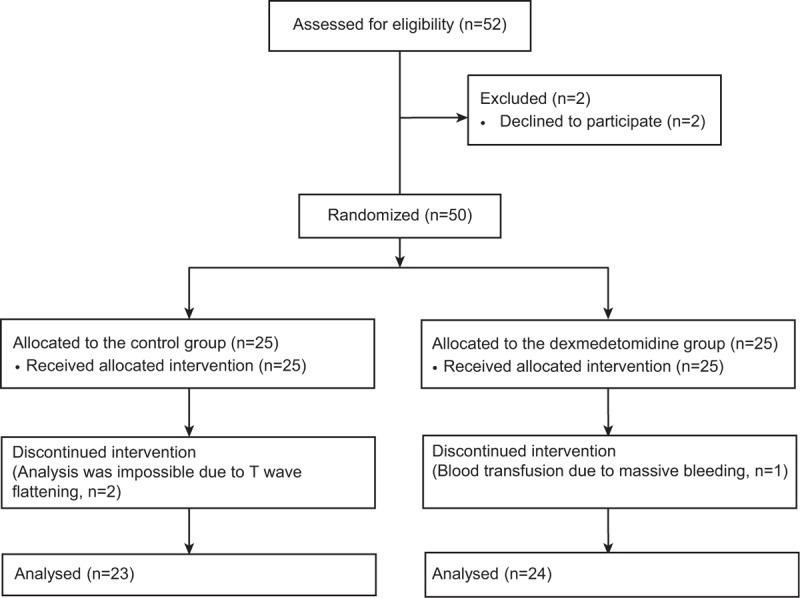
Consort diagram showing the flowchart.

Of the 52 patients assessed for eligibility, 50 were enrolled and randomized into 2 groups, of whom 47 (94%) completed the study (Figure 1). Three patients were excluded from the analysis for the following reasons: analysis was impossible because of T-wave flattening in 2 patients in the control group and red blood cell transfusion due to massive bleeding in 1 patient in the dexmedetomidine group.

The patient characteristics and intraoperative variables, except blood loss and total administered dose of remifentanil, were similar between the 2 groups (Table 1). The total administered dose of remifentanil was lower (548 ± 196 vs 812 ± 346 μg; *P* = 0.003) and blood loss was less (174 ± 110 vs 259 ± 168 μg; *P* = 0.048) in the dexmedetomidine group than in the control group.

**TABLE 1:**
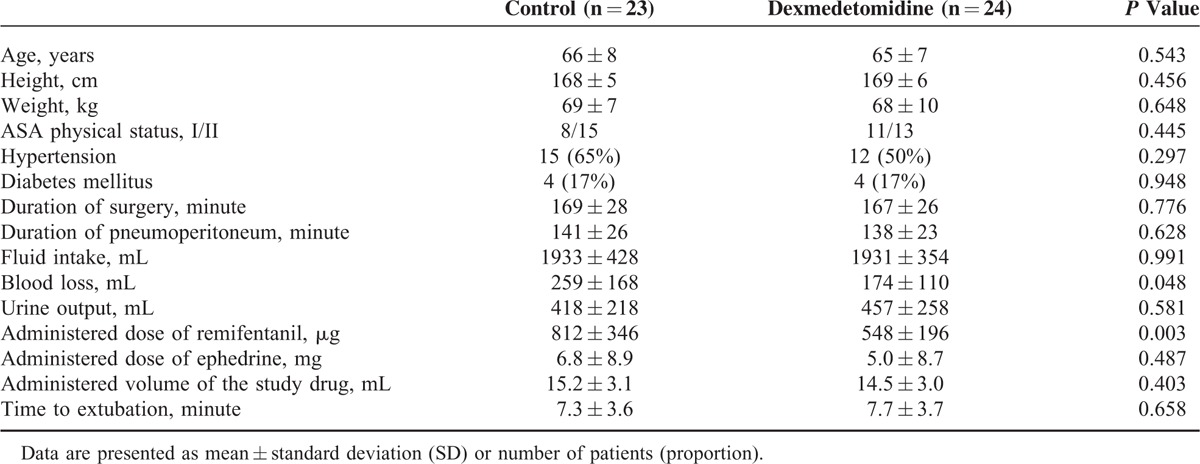
Patient Characteristics and Intraoperative Variables

**FIGURE 2:**
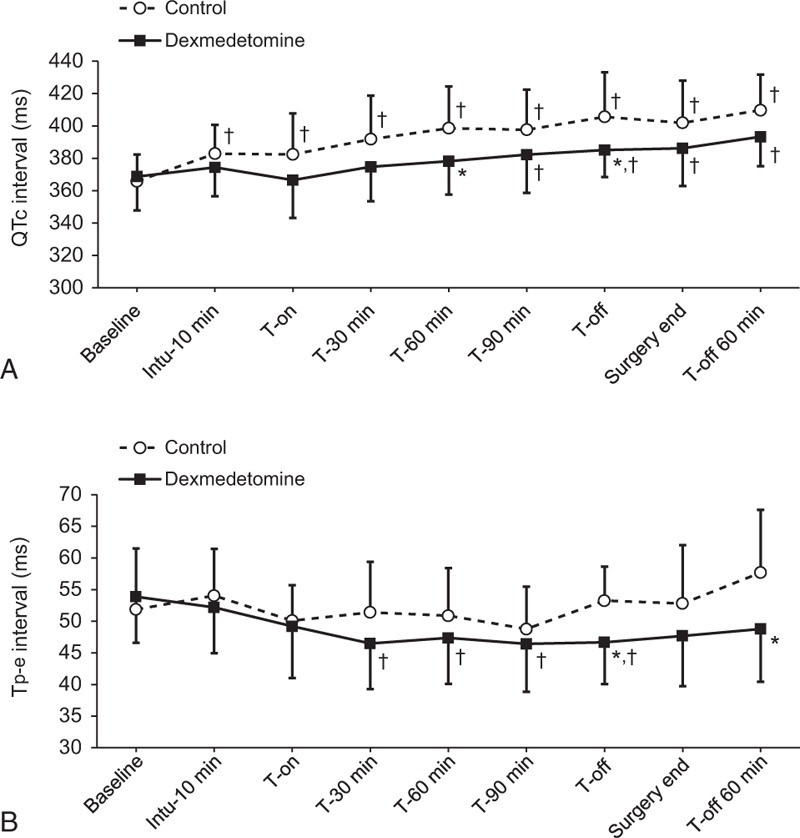
The QTc interval (A) and Tp-e interval (B) from prior induction until 60 minutes after CO_2_ desufflation in the dexmedetomidine and control groups. Baseline = before induction of anesthesia in the supine position, Intu-10 min = 10 minutes after tracheal intubation, T-on = immediately after steep Trendelenburg position with CO_2_ pneumoperitoneum, T-30 min, T-60 min, and T-90 min = 30, 60, and 90 minutes after steep Trendelenburg position with CO_2_ pneumoperitoneum, T-off = immediately after a supine position with CO_2_ desufflation, Surgery end = end of surgery, and T-off 60 min = 60 minutes after a supine position with CO_2_ desufflation in the post-anesthetic care unit. Data are expressed as mean ± SD. ^∗^*P* < 0.05, versus the control group (Bonferroni corrected), ^†^*P* < 0.05, versus the baseline value for each group (Bonferroni corrected). CO_2_ = carbon dioxide, QTc = heart rate-corrected QT, Tp-e = Tpeak–Tend, SD = standard deviation.

The QTc and Tp-e intervals from Baseline until T-off 60 min are presented in Figure 2. Even though the baseline values of QTc and Tp-e interval were similar between the 2 groups, significant intergroup differences were observed overtime (*P*_Group × Time_ = 0.001 and 0.014, respectively). The QTc interval was shorter in the dexmedetomidine group than in the control group at T-60 min (378.2 ± 4.8 vs 398.6 ± 4.9 ms; Bonferroni corrected *P* = 0.039) and at T-off (385.1 ± 4.6 vs 405.6 ± 4.7 ms; Bonferroni corrected *P* = 0.028). The Tp-e interval was shorter in the dexmedetomidine group than in the control group at T-off (46.7 ± 1.2 vs 53. 2 ± 1.3 ms; Bonferroni corrected *P* = 0.005) and T-off 60 min (48.8 ± 1.9 vs 57. 7 ± 1.9 ms; Bonferroni corrected *P* = 0.015). When compared with its baseline value, the QTc interval was significantly prolonged throughout all the time points in the control group, while it was prolonged only after T-90 min in the dexmedetomidine group. In Tp-e interval, no significant change was observed in the control group, but a significant reduction was observed in the dexmedetomidine group during the Trendelenburg position.

None of the patients had a QTc interval of >450 ms before surgery. However, 2 patients in the control group presented a QTc interval of >450 ms, which developed after T-90 min. Twenty-two patients (96%) in the control group and 13 (54%) in the dexmedetomidine group had a QTc interval prolongation of >20 ms from the baseline value during surgery (*P* = 0.001). The maximum QTc interval prolongation from the baseline value during surgery was 46 ± 21 ms in the control group and 24 ± 21 ms in the dexmedetomidine group (*P* = 0.001; Table 2).

**TABLE 2:**
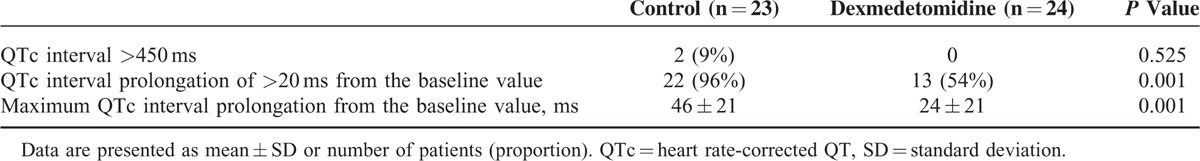
Changes in QTc Interval During Surgery

**FIGURE 3:**
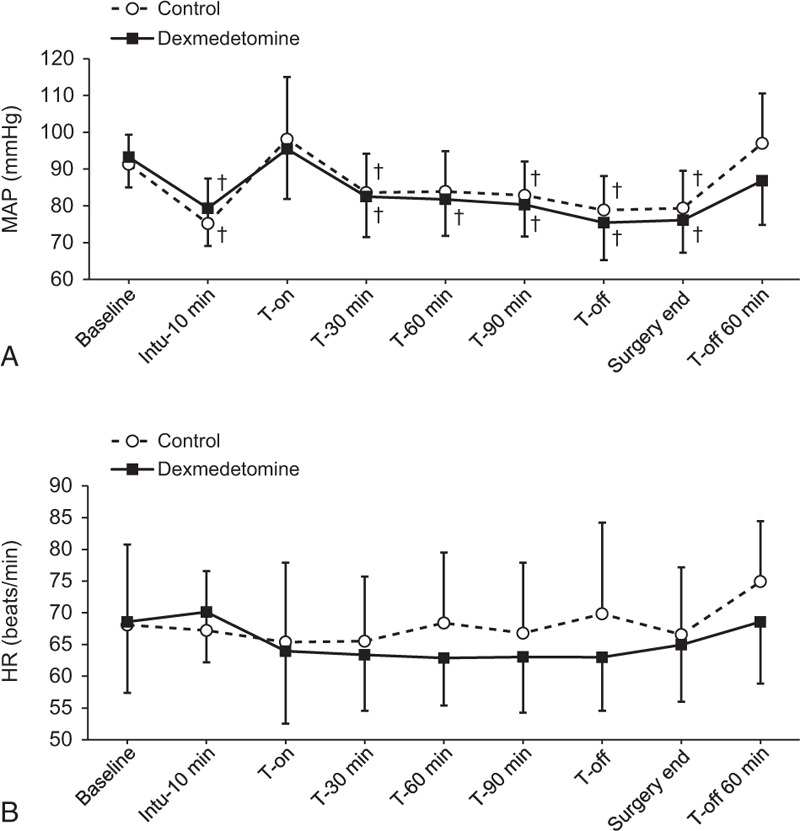
The mean arterial pressure (A), heart rate (B) from prior to induction until 60 min after CO_2_ desufflation in the dexmedetomidine and control groups. Baseline = before induction of anesthesia in the supine position, Intu-10 min = 10 minutes after tracheal intubation, T-on = immediately after steep Trendelenburg position with CO_2_ pneumoperitoneum, T-30 min, T-60 min, and T-90 min = 30, 60, and 90 minutes after steep Trendelenburg position with CO_2_ pneumoperitoneum, T-off = immediately after a supine position with CO_2_ desufflation, Surgery end = end of surgery, and T-off 60 min = 60 minutes after a supine position with CO_2_ desufflation in the postanesthetic care unit. Data are expressed as mean ± SD. ^†^*P* < 0.05, versus the baseline value for each group (Bonferroni corrected). CO_2_ = carbon dioxide, SD = standard deviation.

No significant difference in MAP and HR were observed throughout all the time points between the 2 groups (Figure 3). The total administered dose of ephedrine was also similar between the groups (Table 1), and none of the patients in either group required atropine administration. The EtCO_2_ during surgery was comparable between the 2 groups, and end-tidal sevoflurane concentration was lower in the dexmedetomidine group than in the control group at the end of surgery (*P* = 0.049; Table 3).

**TABLE 3:**
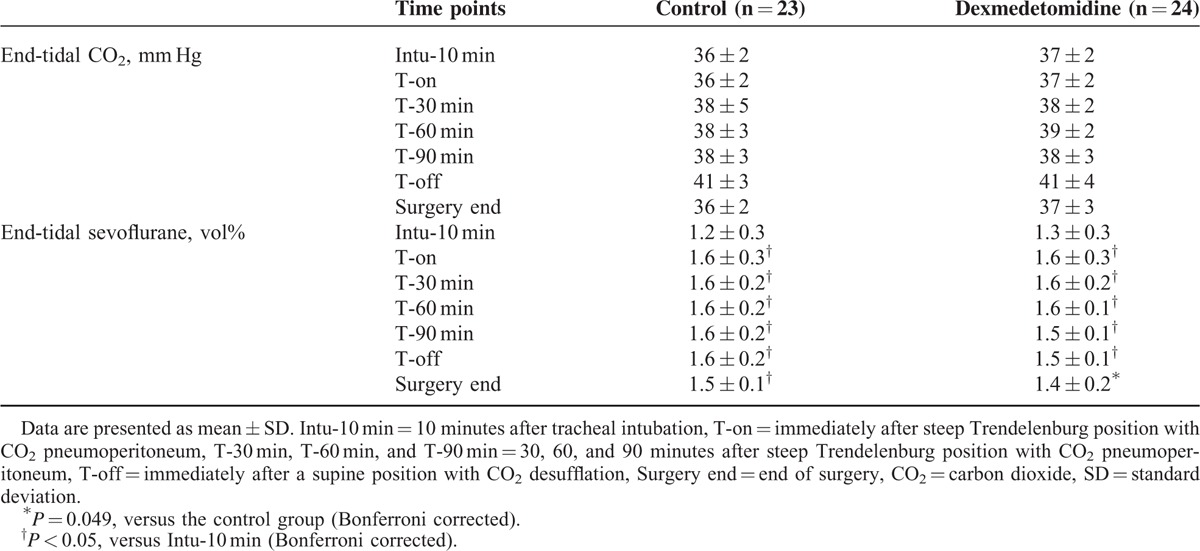
End-Tidal Carbon Dioxide and Sevoflurane Concentrations During Surgery

## DISCUSSION

This prospective randomized, double-blind, placebo-controlled trial was the 1st study regarding the effect of dexmedetomidine on QTc and Tp-e intervals during robot-assisted laparoscopic prostatectomy with steep Trendelenburg position. Dexmedetomidine administration at a rate of 0.3 μg/kg/hour until the end of the Trendelenburg position attenuated the QTc interval prolongation induced by CO_2_ pneumoperitoneum with steep Trendelenburg position. Furthermore, the Tp-e intervals during the Trendelenburg position, when compared with the baseline values, were reduced significantly by dexmedetomidine.

The abnormality of ventricular repolarization, which can be detected on ECG as a prolonged QTc interval, is a well-recognized cause of potentially life-threatening ventricular arrhythmias.^11,12^ Many exogenous factors can lead to QTc interval prolongation, such as medications, electrolyte imbalance, and sympathetic stimulation.^15^ During laparoscopic surgery, sympathetic stimulation can be induced by intraperitoneal CO_2_ insufflation, which leads to increased intraabdominal pressure, hypercarbia, and increased catecholamine and vasopressin.^1–5^ In particular, robot-assisted laparoscopic prostatectomy requires a steep Trendelenburg position with often long insufflation times, which may aggravate sympathetic stimulation and consequently cause the prolongation of QTc intervals.^7^ Robot-assisted laparoscopic prostatectomy has gained popularity during the past decade and widely replaced conventional open prostatectomy in many institutions because it was associated with reduced blood loss, less postoperative pain, and shorter hospital stay.^24^ However, several complications resulted from stimulation of sympathetic activity, such as increased intraocular pressure and significant cardiovascular and respiratory changes.^7,8,25^ In addition, most patients who undergo robot-assisted laparoscopic prostatectomy are elderly with various comorbidities, which may render the patients more vulnerable to abnormalities of ventricular repolarization caused by sympathetic stimulation.^18^ Aging is well recognized to be related with structural and functional changes of the cardiovascular system and torsades de pointes due to QTc interval prolongation, which may develop more easily in patients aged >65 years.^26^ The mean age of the patients in our study was around 65 years. None of our patients had a QTc interval of >450 ms before surgery. However, in the control group, the QTc interval was significantly increased after pneumoperitoneum with steep Trendelenburg position, which led to a QTc interval of >450 ms in 2 patients and a QTc interval prolongation of >20 ms from baseline in 22 patients (96%). As a QTc interval of >450 ms in men is considered prolonged and prolongation of >20 ms from the baseline value can increase the risk of torsade de pointes,^15^ more attention may be needed to prevent or attenuate QTc interval prolongation during robot-assisted laparoscopic prostatectomy, even when preoperative ECG findings are normal. Furthermore, as the QTc interval prolongation did not return to the baseline values until 60 minutes after CO_2_ desufflation, careful observation may be needed even after surgery.

Several trials with opioids, β-adrenergic blockers, or local anesthetics have been conducted to attenuate QTc interval prolongation that results from sympathetic changes during laparoscopy and tracheal intubation. However, results are inconsistent and controversial.^27–32^ Dexmedetomidine, a highly selective α-2 adrenoreceptor agonist, has sympatholytic, sedative, and analgesic properties and does not induce respiratory depression.^19^ In addition to these well-known properties, dexmedetomidine has antiarrhythmic property, and perioperative use of dexmedetomidine was effective in the prevention and treatment of perioperative supraventricular and ventricular arrhythmias after congenital cardiac surgery.^20,21^ Moreover, QTc interval prolongation during tracheal intubation and spinal anesthesia was attenuated by dexmedetomidine administration.^22,23^ Consistent with previous results, dexmedetomidine significantly attenuated the QTc interval prolongation during pneumoperitoneum with steep Trendelenburg position. Significantly fewer patients in the dexmedetomidine group showed a QTc interval prolongation of >20 ms from the baseline value (54% vs 96% in the control group). Compared with the control group, the dexmedetomidine group had a significantly shorter maximum QTc interval prolongation from the baseline (24 ± 21 vs 46 ± 21 seconds). Even though the total administered remifentanil dose was significantly lower in the dexmedetomidine group (548 ± 196 vs 812 ± 346 μg in the control group), the QTc interval prolongation was significantly shorter in the dexmedetomidine group than in the control group. This may imply that dexmedetomidine is more powerful in attenuating QTc interval prolongation than remifentanil, although remifentanil has been known to be effective for attenuating QTc interval prolongation as well.^31,32^

Tp-e interval was measured as a secondary outcome, which reflects the transmural dispersion of ventricular repolarization and has been regarded as a more reliable indicator of risk of torsades de pointes.^13,14^ For Tp-e interval measurement, left precordial leads (V5 and V6) should be selected because they can measure the electrical activity of the left ventricle and reflect the transmural dispersion of repolarization more accurately, whereas bipolar limb leads (I, II, and III) could not detect across the ventricular wall.^33^ Hence, previous studies also used V5 for both QTc and Tp-e interval measurements.^14,17^ The Tp-e interval in the control group was not significantly prolonged during surgery. However, dexmedetomidine significantly reduced the Tp-e interval from its baseline value, which could confirm the antiarrhythmic property of dexmedetomidine. The possible mechanisms for this antiarrhythmic property of dexmedetomidine are as follows: a parasympathomimetic effect that alters the Ca^2+^ current across the myocyte cell membrane, which in turn induces prolonged repolarization, and a central sympatholytic effect.^26^

This study has several limitations. First, whether the effects of dexmedetomidine on ECG changes are dose-dependent remains to be clarified, as we only used a continuous infusion at a rate of 0.3 μg/kg/hour without a loading dose. However, as dexmedetomidine can induce bradycardia and hypotension via sympatholytic effects,^19^ a higher dose may result in hemodynamic instability, especially in elderly patients. Moreover, dexmedetomidine produces transient hypertension when administered as a loading dose.^19^ Thus, a low-dose continuous infusion may be proper when considering patient age and hemodynamic changes during pneumoperitoneum. Second, measurement of HR variability or plasma catecholamine, which would be a useful assessment tool for baseline sympathetic activity, was not performed. However, we could indirectly infer that the sympathetic activities of the patients in the 2 groups were similar, as the baseline HR, MAP, and QTc interval were comparable. Lastly, more than 50% of the patients had hypertension or diabetes mellitus, which can influence the ECG changes during pneumoperitoneum. However, owing to the high prevalence of hypertension and diabetes mellitus in the elderly population, the results of this study could be well attributed to common clinical settings.

In conclusion, continuous infusion of dexmedetomidine at a rate of 0.3 μg/kg/hour until the end of the Trendelenburg position significantly attenuated the QTc interval prolongation induced by CO_2_ pneumoperitoneum with steep Trendelenburg position during robot-assisted laparoscopic prostatectomy. Furthermore, dexmedetomidine reduced the Tp-e interval. Therefore, dexmedetomidine administration may be effective for patients susceptible to the development of ventricular arrhythmia during robot-assisted laparoscopic prostatectomy.
